# Synthesis and characterization of novel zinc–organic framework for the effective removal of Alizarin Red S

**DOI:** 10.1038/s41598-025-29056-5

**Published:** 2025-12-08

**Authors:** Mohamed A. Abdelwahab, Abdelhamid M. Abdelhamid, Ayman S. Eliwa, Ashraqat M. Abdelhamid, Sohaila Abdelhady, Soad Auf, Hala A. Awad, Raghad M. Abdelkader, Jana Ghanem, Malak H. Mohamed, Mariam Gamaleldin, Saher A. Ali, Gehad G. Mohamed, Maha Alhelf

**Affiliations:** 1 Chemistry Department, Faculty of Science, Cairo University, Giza, 12613 Egypt; 2 Biotechnology School, Nile University, Giza, 12588 Egypt; 3 Nanoscience Department, Faculty of Basic and Applied Sciences, Egypt-Japan University of Science and Technology, New Borg El Arab, Alexandria, 21934 Egypt; 4 Medical Biochemistry and Molecular Biology Department, Faculty of Medicine, Cairo University, Cairo, Egypt

**Keywords:** Zn-MOF, Alizarin red s dye, XRD, SEM, BET, Pseudo-second order reaction, Langmuir model, Catalysis, Electrochemistry, Inorganic chemistry, Materials chemistry

## Abstract

Water treatment has become a pressing global issue due to the increasing number of pollution sources. Among the major contributors to water contamination is the widespread use of dyes, such as Alizarin Red S, across various industries. In this study, Zn-MOF nanoparticles were prepared using the conventional solvothermal method and characterized through Fourier-transform infrared (FT-IR) spectroscopy, X-ray diffraction (XRD), scanning electron microscope coupled with energy dispersive X-ray spectroscopy (SEM-EDX), Brunauer-Emmett-Teller (BET) analysis as well as thermogravimetric analysis (TGA). The adsorption performance of Zn-MOF nanoparticles for removing Alizarin Red S (ARS) was investigated using batch adsorption experiments. Key parameters, including Zn-MOF dosage, initial ARS concentration, pH and contact time were evaluated under ambient conditions. The results demonstrated an ARS removal efficiency of approximately 71% at pH 4, a Zn-MOF dosage of 1 g/L and a primary ARS concentration of 20 ppm within 40 min. Additionally, the material exhibited reusability for up to three cycles, maintaining a removal efficiency of 60%. Freundlich, Langmuir, and Temkin were three isotherm and kinetic models applied to illustrate the adsorption mechanism. Adsorption was found to align with the Langmuir isotherm model as well as following pseudo-second-order kinetics.

**Supplementary Information:**

The online version contains supplementary material available at 10.1038/s41598-025-29056-5.

## Introduction

Water, a vital resource, plays a crucial role in ecosystems and serves as an essential component in agricultural and industrial activities. Its availability and quality directly affect the well-being of all living organisms^1^. Synthetic dyes are extensively utilized for having vibrant colors and high chemical stability in multiple sectors such as textiles, paper, and plastics, which makes them valuable but difficult to manage sustainably^2^. Emancipation of these dyes into water bodies has become a significant environmental concern, as their complex aromatic structures^3^ enhance chemical stability while hindering natural degradation processes, leading to long-term environmental persistence. These pollutants disrupt aquatic ecosystems by obstructing sunlight, reducing photosynthetic activity, and causing toxic effects on aquatic organisms through bioaccumulation and prolonged exposure to hazardous chemicals^4^. As the dye-dependent industry continues to expand alongside the rising need for textiles and garments, the demand for dyes has been increasing proportionally since then. As a result, large amounts of synthetic dyes have built up in the environment. These dyes are widely employed in other sectors in addition to the textile industry, such as paper, food, rubber, and cosmetics. The occurrence of these dyes in industrial wastewater is very apparent, and the oxidation products of these pigments are often carcinogenic, even at very low concentrations. Additionally, synthetic dyes pose many health risks to other living organisms alongside humans, causing mutations, cytotoxicity, neurotoxicity, hypersensitivity, and mitochondrial toxicity. Many countries have tightened the environmental laws governing the textile sector as a result of these arising concerns^5,6^. Organic dyes represent prevalent contaminants found in wastewater across multiple industries, with significant contributions from textile dyeing (54%), paper production (10%), tanneries (8%), dye manufacturing (7%), and other sectors (21%). Annually, approximately 700,000 tons of dyes are produced commercially. Dyes often showcase resistance to external surrounding factors such as temperature and light, in addition to chemical agents, which increase their solubility in water. While textile wastewater typically contains dye concentrations around 300 mg/L, levels as low as 1 mg/L are detectable and pose significant environmental and health risks. Different dye types, ranging from acidic, basic, reactive, and direct, intensify the complexity of dye removal from wastewater as they co-occur with each other, with the highest toxicity levels attributed to the basic and direct diazo dyes^7^. Among synthetic dyes, Alizarin Red S (ARS) presents a significant challenge due to its high-water solubility and associated health and environmental risks. ARS is an azo dye commonly used for dyeing wool and nylon. Similar to Alizarin Yellow R (AYR), ARS is highly soluble in water, exhibits considerable toxicity, and has been identified as a potential carcinogen, posing severe threats to aquatic ecosystems^8^. Its impact on water contamination is particularly concerning, as it is classified as an acutely toxic dye and a carcinogenic substance. Ingestion of ARS is highly hazardous, and exposure can cause severe eye irritation as well as skin-related effects, including redness, itching, and inflammation^9^. Conventional water treatment methods include electrochemical techniques, degradation, chemical reduction^10,11^, membrane filtration^12,13^, anaerobic and aerobic treatments, coagulation, flocculation^12^, and adsorption^14^. Among these, adsorption remains the most widely used approach for addressing water contamination^7^. However, traditional methods often face limitations such as high costs, large equipment requirements, and susceptibility to rapid contamination. Therefore, exploring innovative and more effective treatment strategies has become essential^15^. Adsorption has drawn notable attention in recent years due to its efficiency, stability, and minimal byproduct formation. The process involves trapping pollutants through either physical interactions or chemical bonding, with its effectiveness largely influenced by the surface area, active sites, and selectivity of the porous adsorbent used^16^. MOF, also known as Metal-organic frameworks, are highly porous materials composed of metal ions or clusters coordinated with organic linkers, forming a cage-like structure with a large surface area and tunable pore sizes^17,18^. This distinctive architecture grants MOFs exceptional adsorption capacity, thermal stability, and chemical versatility, enabling effective interactions with dye molecules through mechanisms such as electrostatic attraction, π–π stacking, and hydrogen bonding. These properties make MOFs highly suitable for applications including dye removal, gas storage, and catalysis^19,20^. Additionally, these interactions make the removal of a wide range of organic dyes from water sources an easier process^21^. For example, Fe-BTC, an iron-based MOF, has demonstrated significant efficiency in adsorbing toxic dyes from wastewater due to its strong binding interactions and stability. Its environmentally friendly synthesis, commonly carried out at ambient temperature with water serving as the solvent, further enhances its suitability for large-scale, sustainable wastewater treatment. Similarly, zinc-based MOFs (Zn-MOFs), which consist of zinc ions coordinated with organic linkers^22^, have emerged as an alternative for removing organic dyes from water. Their high porosity and chemical stability contribute to their strong adsorption performance, which is affected by parameters like pH levels and the concentration of the dye. Optimizing these parameters enhances dye removal efficiency^23^. Furthermore, Zn-MOFs are favored for their simple synthesis process, making them scalable and cost-effective for wastewater treatment applications^24^. This study aims to synthesize Zn-MOF nanomaterials using the conventional solvothermal method and evaluate their structural characteristics, surface area, and thermal stability. Additionally, their effectiveness as adsorbents to investigate the removal efficiency of Alizarin Red S (ARS) in wastewater treatment is evaluated. Key parameters affecting the adsorption process were analyzed and optimized, and the kinetic and isotherm models governing the adsorption mechanism were identified.

## Experimental

### Materials and methods

In this study, Analytical-grade reagents and distilled water were utilized throughout all experimental processes. The reagents listed below were purchased from Sigma-Aldrich: zinc acetate, terephthalaldehyde (1,4-benzenedicarbaldehyde) purity of 98%, anthranilic acid (2-aminobenzoic acid) purity of 98%, hydrochloric acid (HCl) purity of 98%, sodium hydroxide (NaOH) purity of 98%, and calcium chloride (CaCl₂) purity of 98%,. Additionally, absolute anhydrous ethanol, which was spectroscopically pure, was used. All glassware was thoroughly cleaned, and distilled water was collected and used in all experimental preparations.

#### Schiff base ligand (H_2_L) linker synthesis

**Fig. 1:**
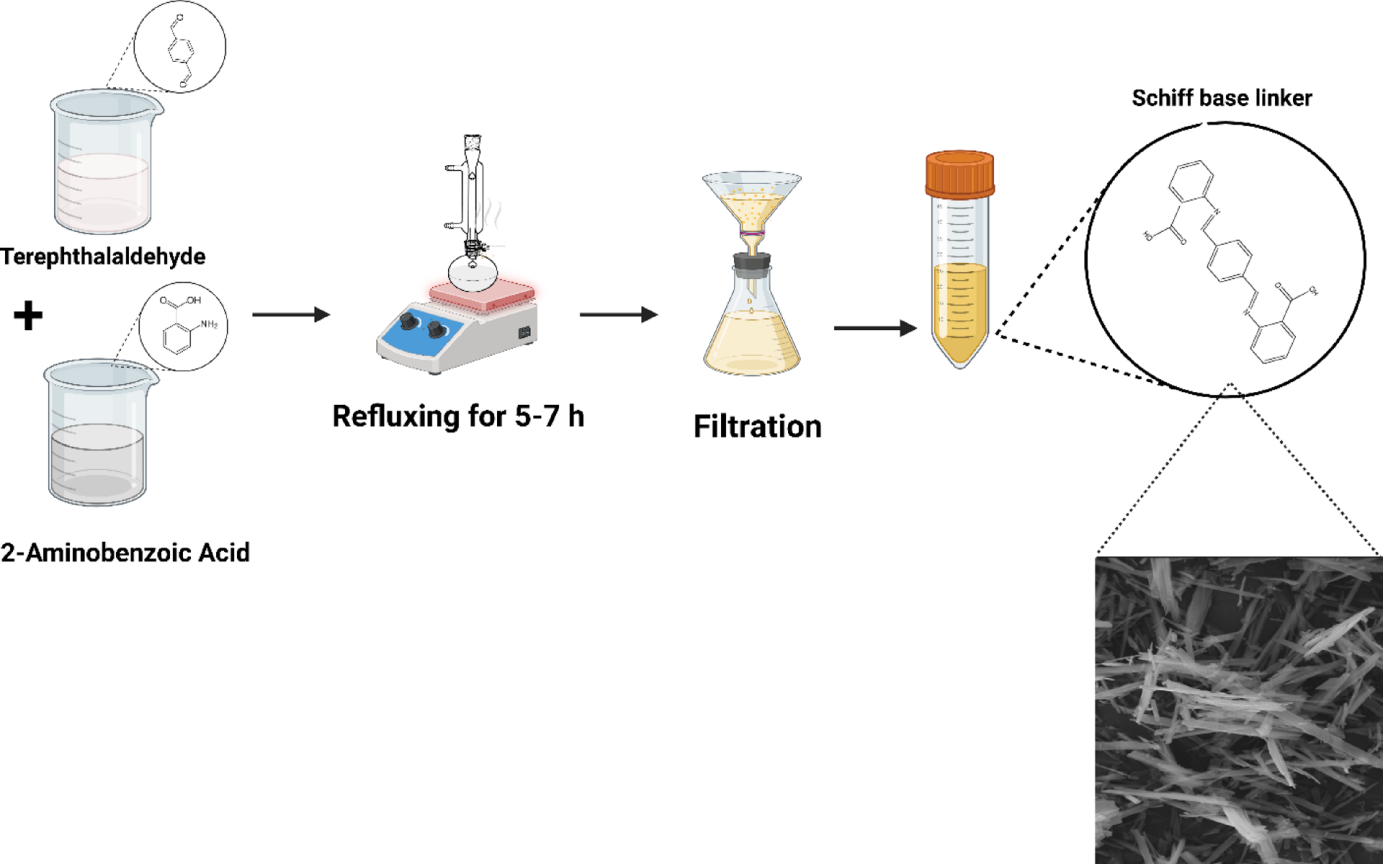
Synthesis of Schiff base ligand (H_2_L).

Figure 1 shows that our Schiff base ligand (H_2_L) was synthesized using a 1:2 molar ratio of terephthalaldehyde (1,4-benzenedicarbaldehyde) (1.04 g) and anthranilic acid (2-aminobenzoic acid) (2.13 g) was dissolved in a saturated ethanolic solution. The mixture was thermally stirred until ligand formation was complete. Cold ethanol was used to wash the produced ethanol thoroughly and the filtrate became clear after being filtered multiple times. Consequently, the solid ligand was dried then using anhydrous calcium chloride in a dehydrator.

#### Conventional hydrothermal synthesis of novel Zn-MOF

**Fig. 2:**
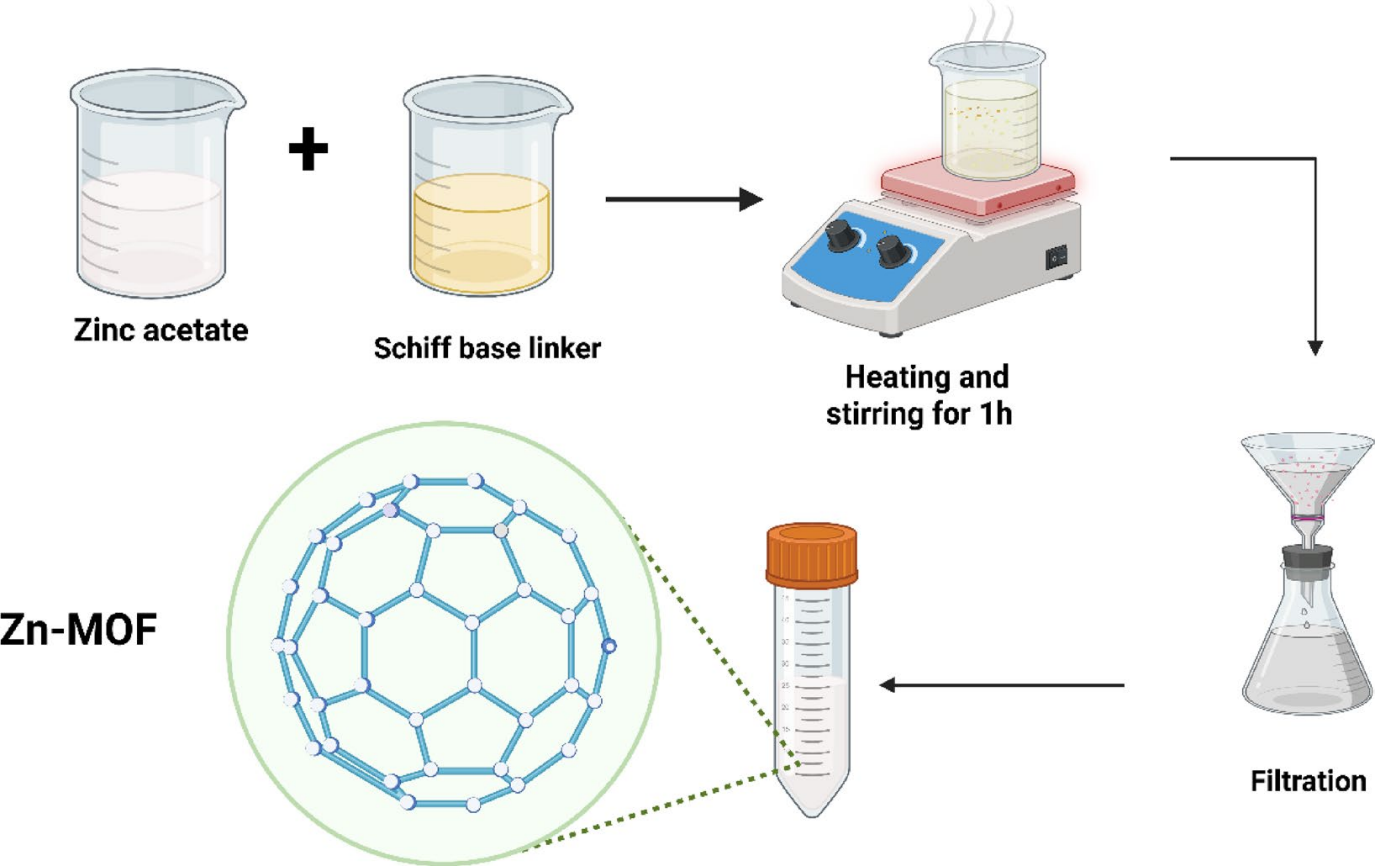
Novel Zn-MOF synthesis.

For the Zn-MOF complex to be synthesized, 50 mL of absolute ethanol was used to dissolve the Schiff base ligand (H_2_L; 1.376 g, 3.7 mmol). Separately, to prepare the solution, dissolution of zinc acetate was carried out using 30 mL of ethanol (0.81115 g, 3.7 mmol), maintaining a 1:1 molar ratio with H_2_L. Consecutively, for 3 h, the two solutions were refluxed and blended. After the reaction, the mixture underwent centrifugation to separate the product, which was then washed three times initially with 50 mL of water, followed by 10 mL of ethanol as shown in Fig. 2. The resulting sample was dried at 130 °C for 12 h and then allowed to cool at room temperature in air.

### Measurements and instruments

A Perkin-Elmer 1650 spectrometer with the range of 4000–400 cm⁻¹ with KBr pellets was used to obtain FT-IR (Fourier-transform infrared) spectra. The PXRD (powder X-ray diffraction) pattern was studied at the EGNC (Egypt Nanotechnology Center) using a Bruker D8 Discover X-ray diffractometer (Bruker AXS Inc., 35 kV, 30 mA). The BET (Brunauer-Emmett-Teller) pore size and surface area distribution were calculated through nitrogen adsorption-desorption analysis at 77 K. Before conducting adsorption tests, the material was degassed under high vacuum for 4–12 h. The Nova Touch LX2 analyzer was used to perform the BET analysis. Scanning electron microscope (SEM) images were captured utilizing a Quanta 250 FEG model with an EDX (energy-dispersive X-ray) unit for compositional analysis. The SEM operated at an accelerating 30 kV of voltage, magnifying up to 60,000×. A Shimadzu TG-50 H thermal analyzer was used to assess thermal stability, with measurements conducted up to 1000 °C starting from room temperature at a heating rate of 5 °C/min. The thermal analysis revealed an initial weight loss of 32.7% between 12.4 and 23.3 °C. A Shimadzu UVmini-1240 UV-Vis spectrophotometer was used to record UV-Vis spectra.

### Adsorption studies

A stable temperature of 25 ± 1 °C was maintained during the adsorption experiments. A dye solution (20 mg/L) with a volume of 50 mL was treated using 0.05 g of adsorbent. pH levels were regulated to reach the target level using 0.1 M solutions of either NaOH or HCl, as required.

**Fig. 3:**
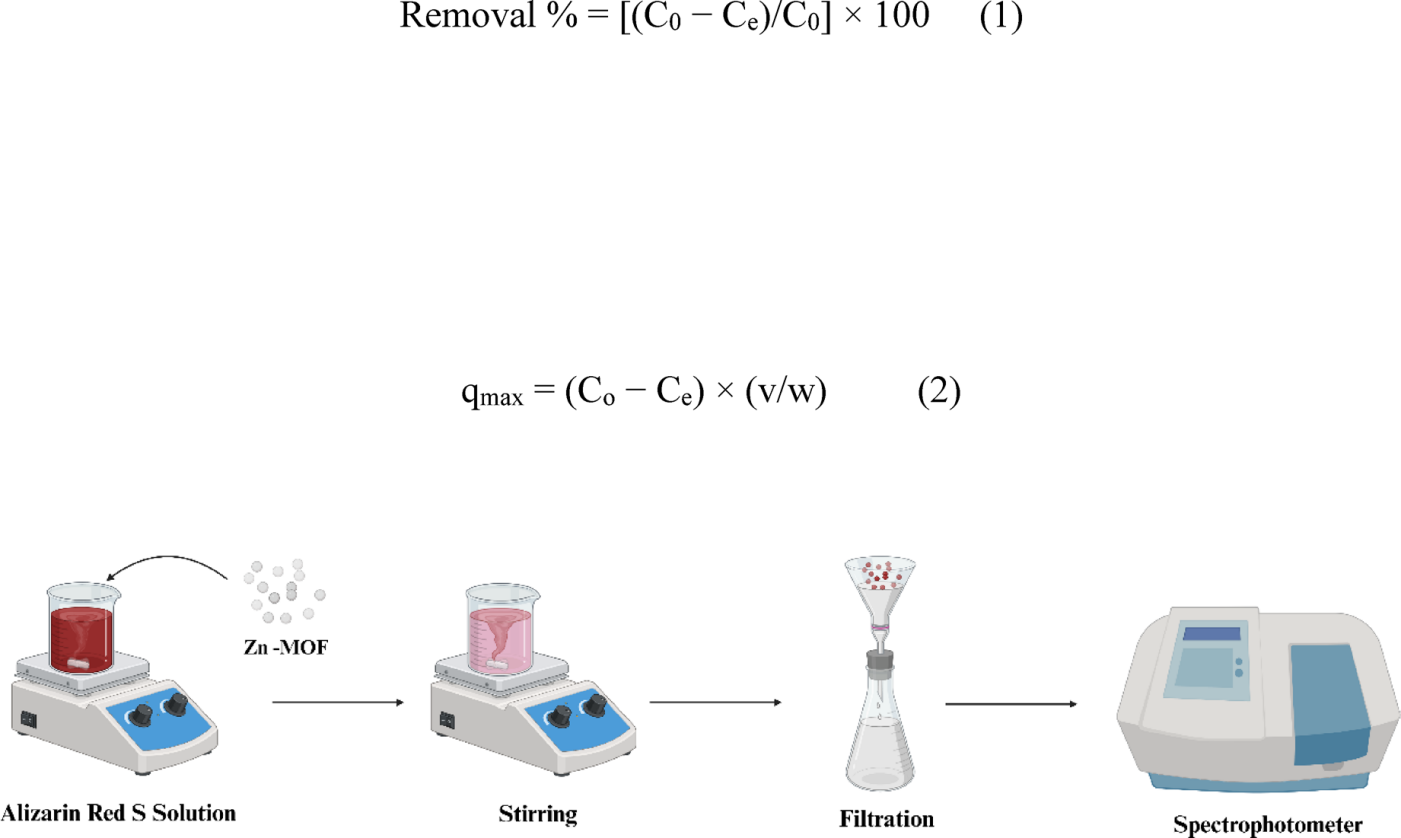
ARS removal using Zn-MOF.

Following the specified adsorption time, Filtration was used to remove the adsorbent from the samples through a 45 μm polyethylene membrane as shown in Fig. 3. To ensure reliable results, all experiments were conducted in triplicate. The ARS concentration in the filtrates was quantified at predetermined time intervals using a UV- Vis spectrophotometer (UVmini- 1240, Shimadzu) at a wavelength of 326 nm. Several parameters were examined, including the pH value, Starting dye concentration and exposure duration and adsorbent dosage. The percentage of dye removal was calculated using Eq. (1):

1 $${Removal}\:{\%}=[(C_0-C_e)/C_0]\times100$$

C₀ denotes the initial dye concentration (mg/L), while Cₑ refers to the equilibrium concentration following adsorption (mg/L), V denotes the volume of the solution (L), and w indicates the mass of dry adsorbent used (g). Additionally, the adsorption capacity of the adsorbent, expressed as (mg of dye/gram of dry adsorbent), was determined using Eq. (2):

2 $$q_{max}=(C_0-C_e)\times(v/w)$$

## Results and discussion

### Characterization of schiff base ligand

**Fig. 4:**
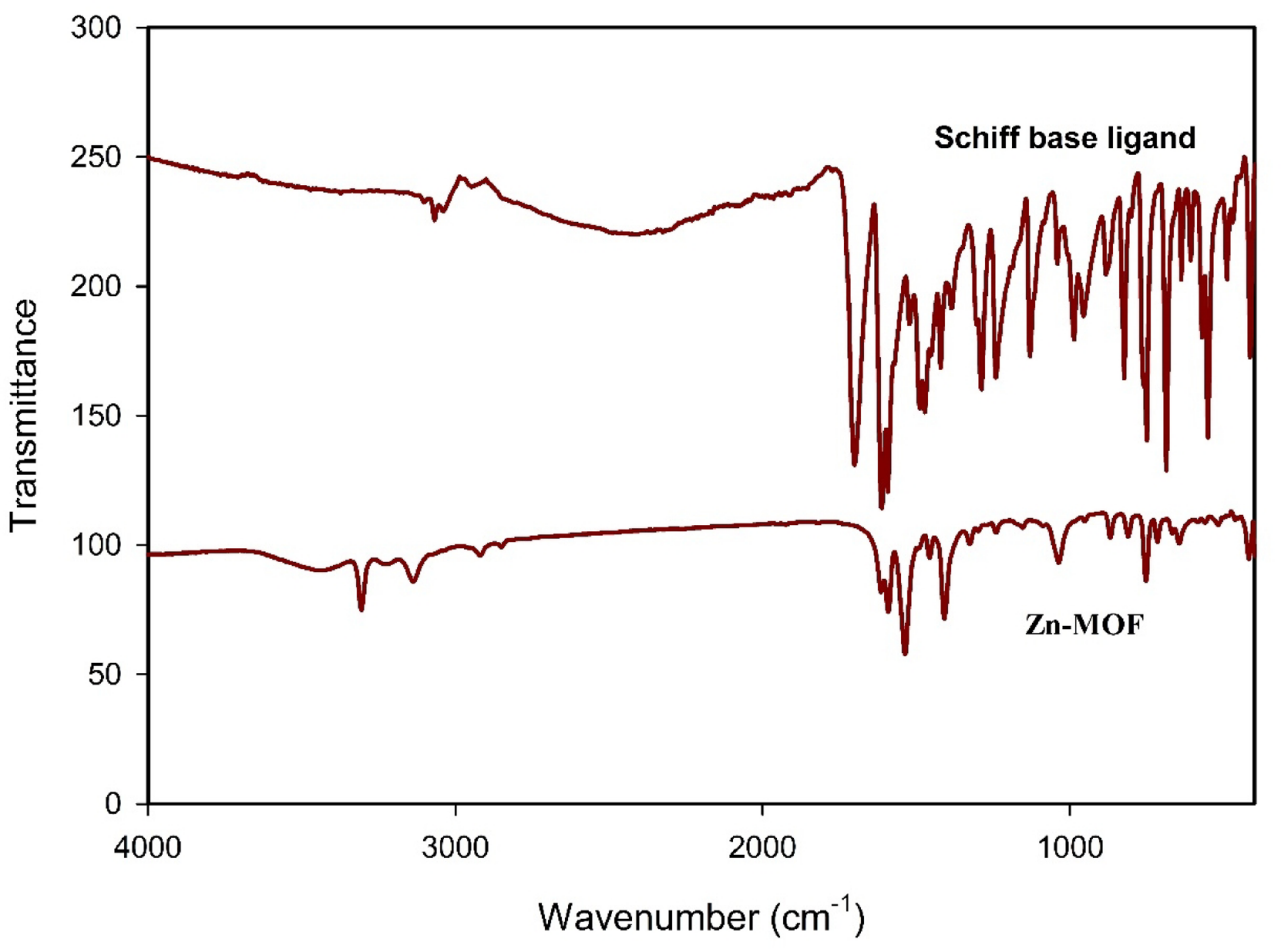
FT-IR spectrum of the synthesized Schiff base ligand (H_2_L) and Zn-MOF.

A stable and well-structured metal-organic framework (MOF) requires an effective organic ligand for its formation. As part of this investigation, a Schiff base ligand was synthesized via a condensation reaction between 2-aminobenzoic acid and benzene-1,4-dicarbaldehyde, yielding a yellow precipitate. The successful formation of the ligand was confirmed using FT-IR (Fourier-transform infrared spectroscopy), as shown in Fig. 4. Absence of the NH_2_ stretching band indicated that the reaction was complete, while a new azomethine (-CH = N) band appearing at 1613 cm⁻¹ confirmed Schiff base formation^25,26^. Additionally, the presence of asymmetric (-COO⁻) and symmetric (-COO⁻) stretching bands at 1422 cm⁻¹ and 1386 cm⁻¹^17^, respectively, further supported the structural integrity of the ligand. The ligand was found to be soluble in ethanol-water and dimethylformamide (DMF) and displayed high thermal stability, making it suitable for use in MOF synthesis (Fig. 1).

### Characterization of Zn-MOF

#### FT-IR analysis

The Schiff base ligand synthesized was used as the organic linker in the preparation of Zn-MOF^27^. FT-IR spectroscopy was employed to analyze its coordination with zinc ions, as depicted in Fig. 4. The results revealed that the carboxylate (-COO⁻) stretching vibrations had shifted, confirming metal coordination. Also, the appearance of the symmetric and asymmetric stretching bands of the carboxylate group at 1668 cm^− 1^ and 1421 cm⁻¹, respectively, with a bidentate chelating coordination mode^27,28^. Furthermore, the presence of a Zn–O stretching band at 515 cm⁻¹ provided additional evidence of the successful incorporation of zinc into the framework.

#### Powder X-ray diffraction pattern (PXRD)

**Fig. 5:**
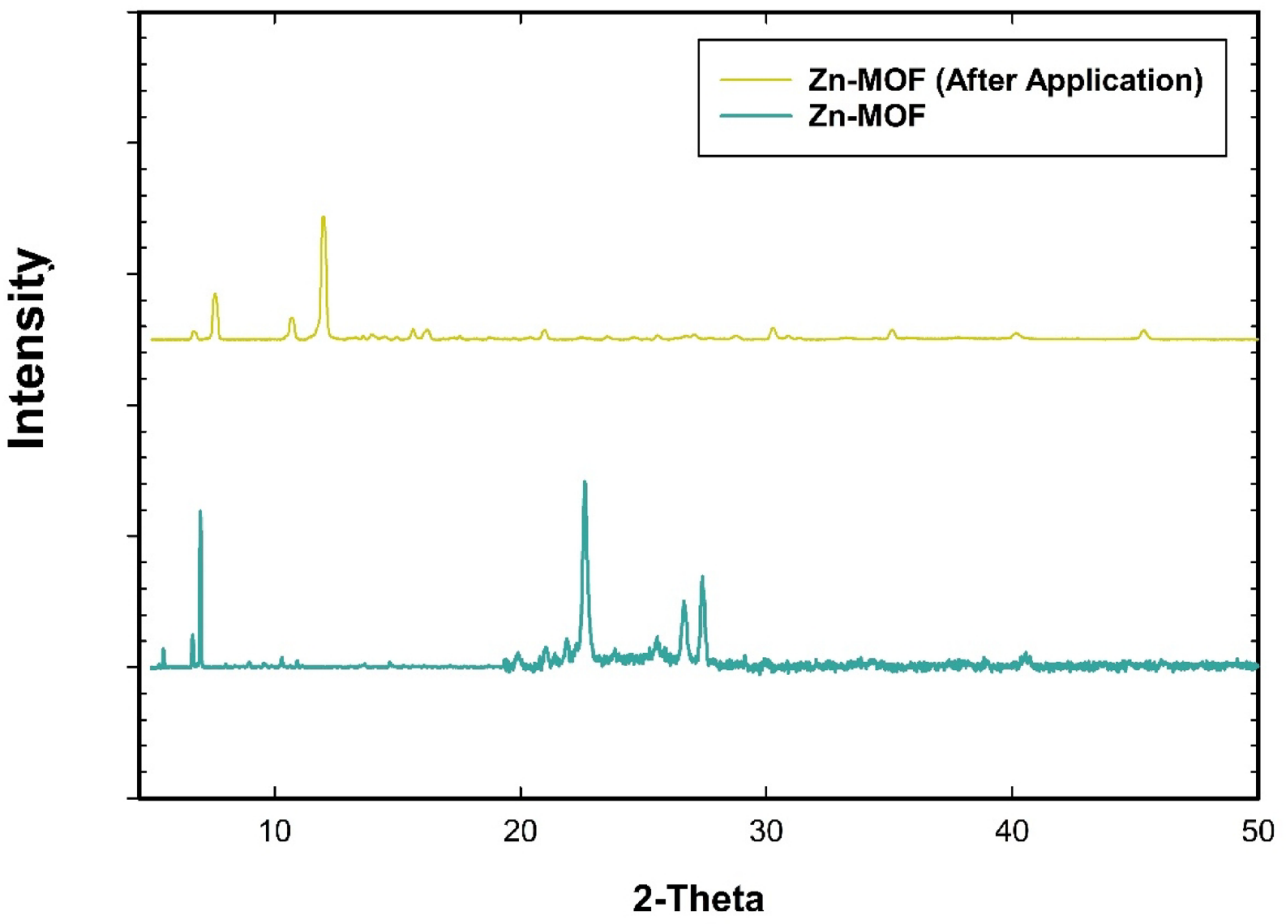
Powder X-ray diffraction patterns of Zn-MOF before and after the application.

The crystallinity and structural integrity of Zn-MOF were evaluated using PXRD analysis, as shown in Fig. 5. The obtained diffraction pattern exhibited sharp and well-defined peaks at 6.87^o^, 11.5^o^, 12.6^o^, 12.7^o^, 20.3^o^, 25.4^o^ and 27.8^o^. The obtained diffraction pattern exhibited sharp and well-defined peaks, closely matching previously reported Zn-MOF structures. This result confirmed that the synthesized Zn-MOF possessed high crystallinity and an ordered framework, which is crucial for its functional applications in catalysis and adsorption. Notably, after applying the material in the application, most of the characteristic diffraction peaks remained at their original positions, and the crystallinity was retained, despite a decrease in the intensity of certain reflections (Fig. 5).

#### BET surface area analysis

**Fig. 6:**
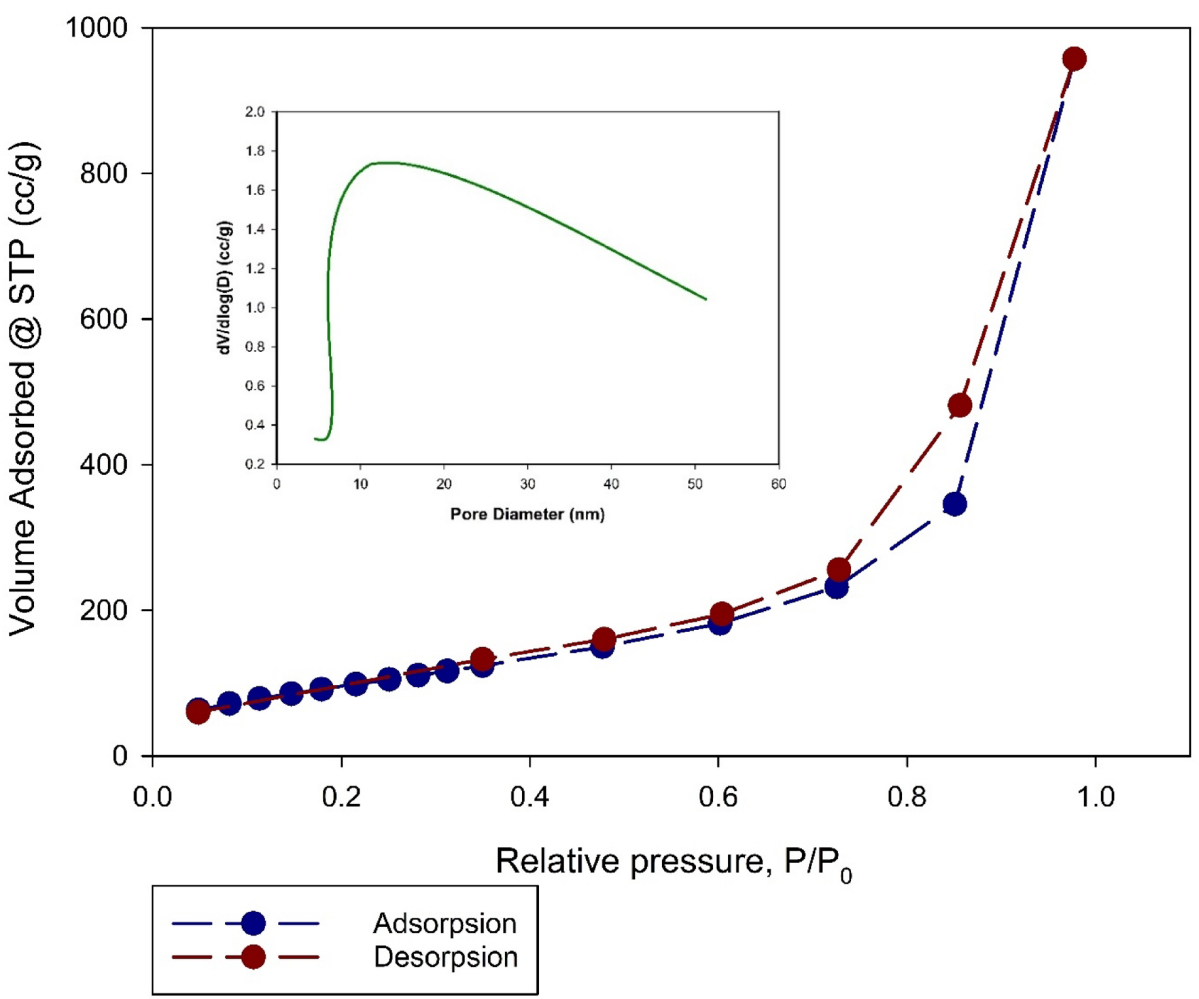
Adsorption–desorption isotherm for synthesized Zn-MOF and BJH pore size distribution diagram.

Brunauer–Emmett–Teller (BET) analysis was employed to determine the surface area and porosity of the Zn-MOF, with the results illustrated in Fig. 6. The nitrogen adsorption-desorption isotherms displayed a type IV pattern, indicative of mesoporous structures^30^. The Zn-MOF demonstrated measured textural properties include a BET surface area of 430 m²/g, a pore volume of 2.27 cm³/g, and an average pore diameter of 4.61 nm. These findings confirmed the material’s high porosity, making it suitable for gas storage, adsorption, and catalytic applications as how it is shown in (Fig. 6).

#### SEM image and EDX analysis of Zn-MOF

**Fig. 7:**
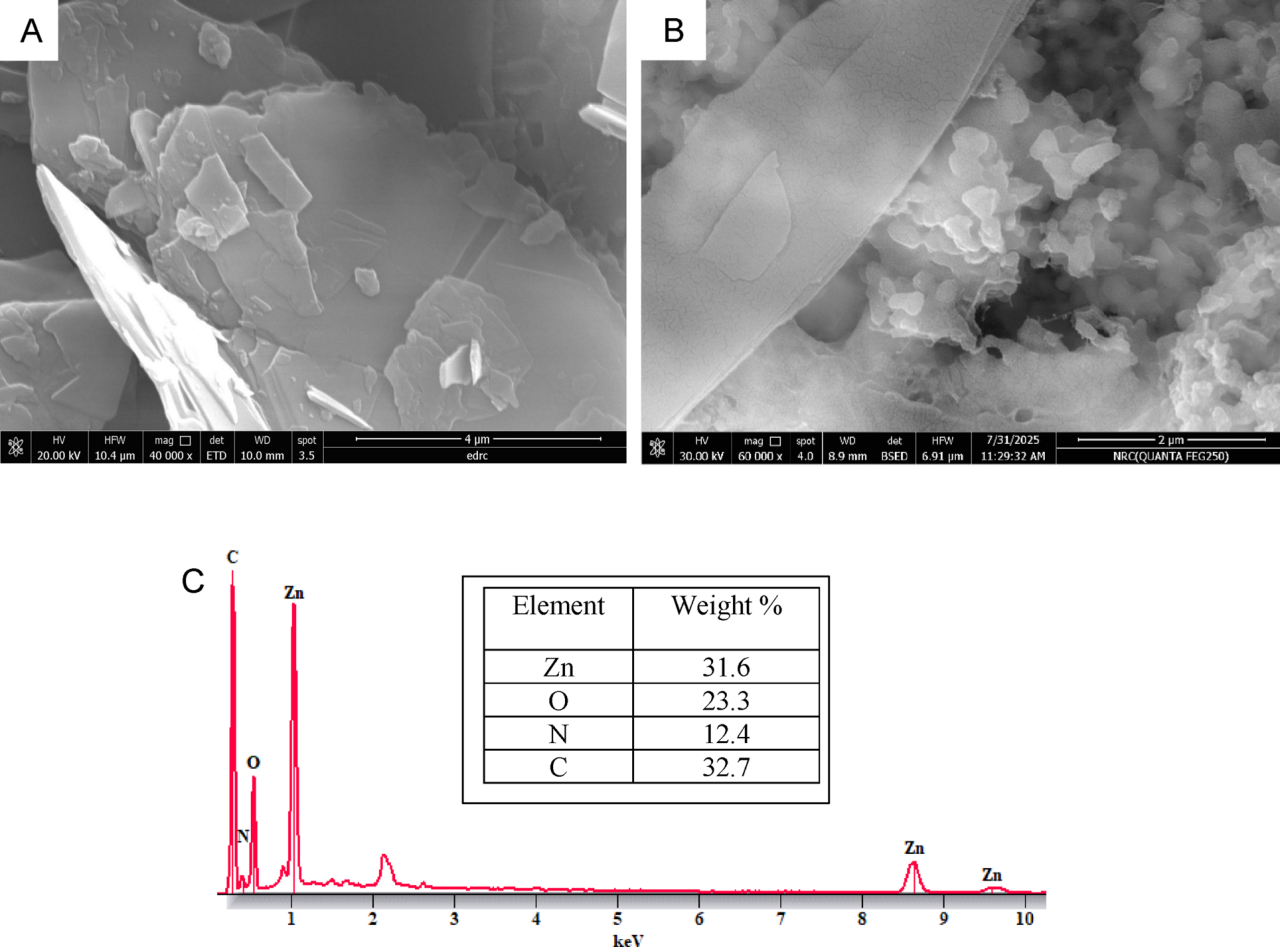
**(A)** SEM image of Zn-MOF, (**B)** SEM image of Zn-MOF after the application and (**C)** EDX spectrum of Zn-MOF.

The morphological characteristics and elemental analysis of Zn-MOF were investigated using Surface morphology and elemental composition were investigated using SEM and EDX, respectively, as shown in Figs. 7. SEM imaging revealed that the Zn-MOF particles were nano-sized, with dimensions ranging from 36 nm to 97 nm, confirming a uniform and well-defined structure. SEM analysis further supported the structural stability of the Zn-MOF, showing that the particle morphology remained virtually unchanged after application. EDX analysis revealed that the main elements present were zinc (Zn), carbon (C), nitrogen (N), and oxygen (O), with their composition ratios aligning with the expected molecular framework. These results confirmed the formation of Zn-MOF and highlighted its potential applicability as both a catalyst and an adsorbent, based on the presence of active sites.

#### Thermal analysis

**Fig. 8:**
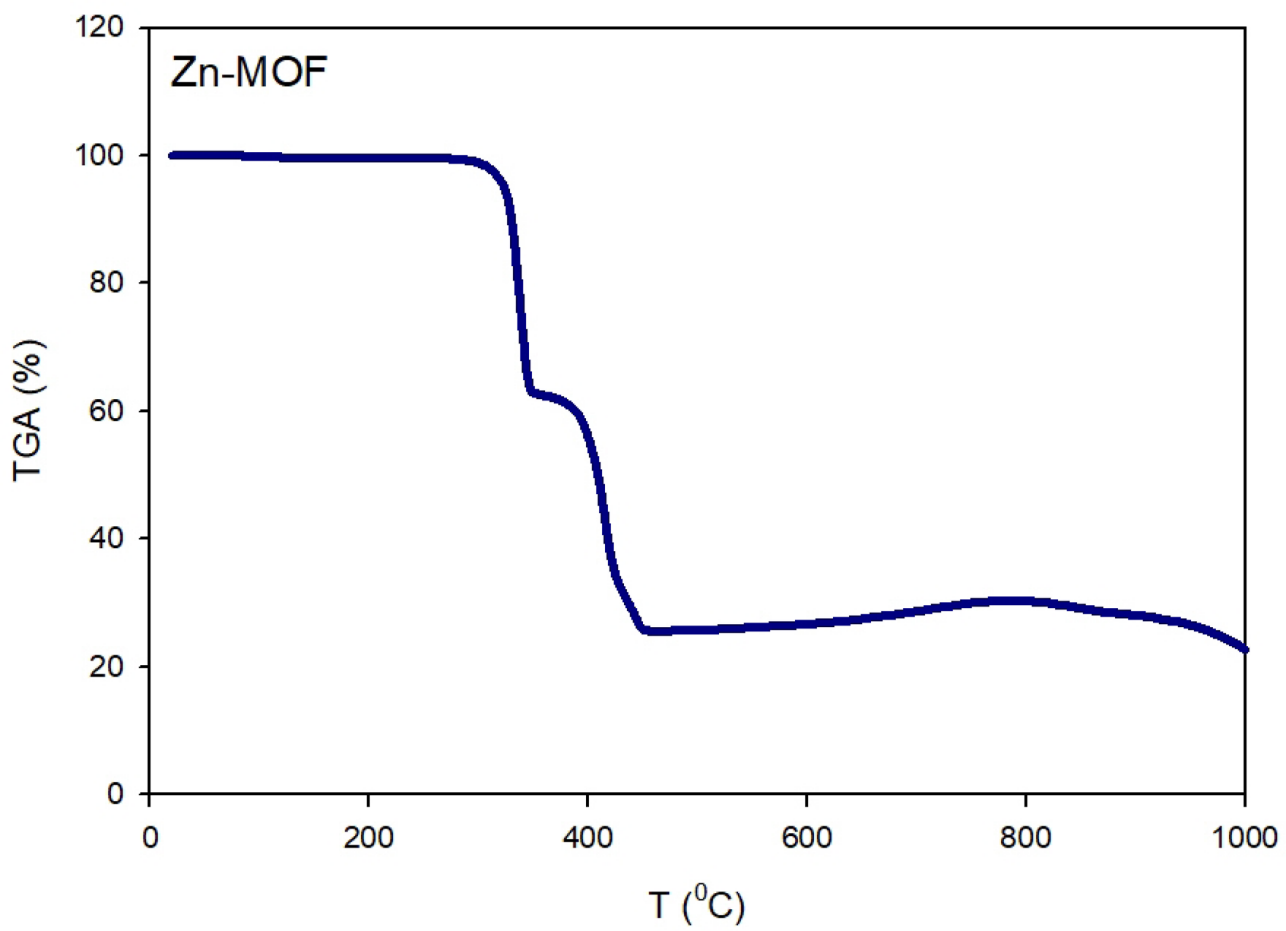
Thermal decomposition profile of Zn-MOF using TGA analysis.

To evaluate the stability of Zn-MOF thermally, thermogravimetric analysis (TGA) was performed, and the results demonstrated in Fig. 8. The decomposition of Zn-MOF occurred over three sequential stages. In the first stage (30–160 °C), a 0.5% reduction in weight was detected, this was ascribed to the evaporation of residual solvents. The subsequent stage, occurring between 300 and 346 °C, resulted in a 36.5% weight reduction, likely due to the degradation of the organic ligand. In the final decomposition stage (390–450 °C), the material lost an additional 35% of its weight, with a minor weight reduction (6%) continuing up to 1000 °C. The last stage of weight loss indicates the formation of ZnO, confirming that Zn-MOF exhibited high thermal stability. These findings indicate that Zn-MOF is well-suited for use in high-temperature conditions, including gas adsorption, catalytic processes, and environmental cleanup and restoration, as shown in Fig. 8.

#### Investigating the variables influencing the adsorption process

For the assessment of the optimal parameters for efficient ARS dye removal, various factors including pH, initial dye concentration, adsorbent dosage, contact time, and stirring were examined to ensure an effective adsorption process.

#### Effect of pH

**Fig. 9:**
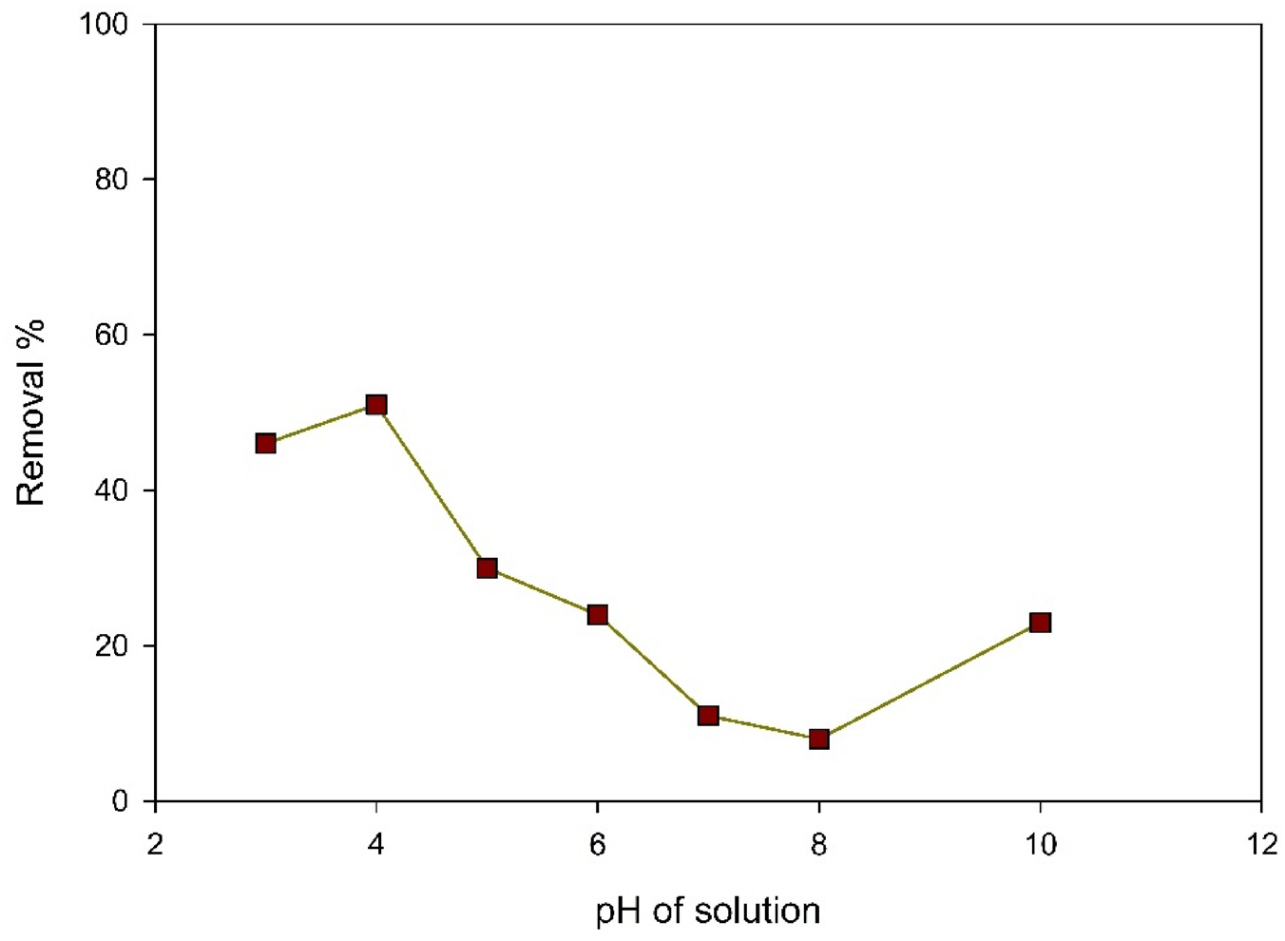
Effect of pH on the elimination of ARS using 0.01 g Zn-MOF/50 mL, 40 ppm dye concentration, and 40 min.

**Fig. 10:**
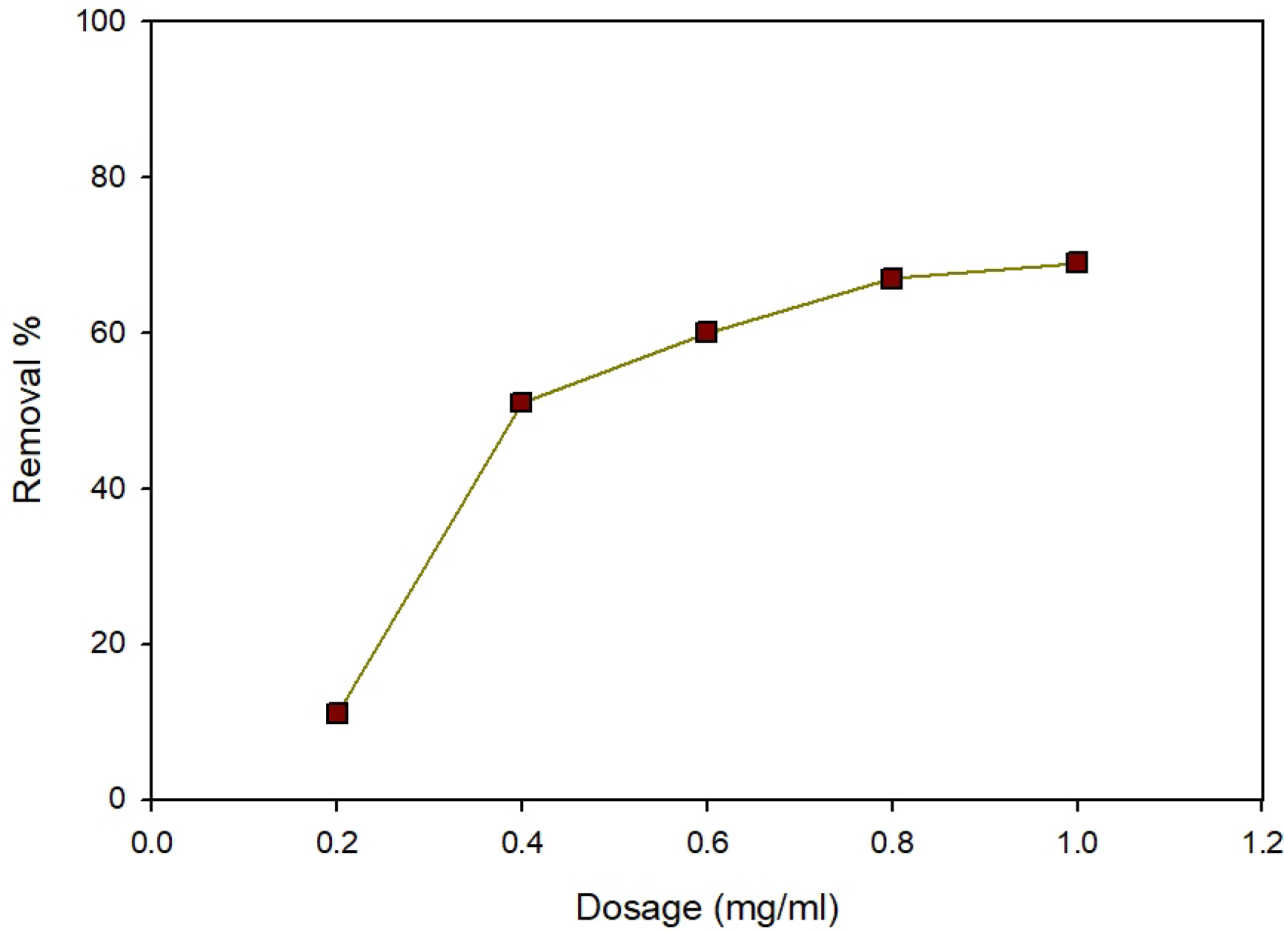
Effect of adsorbent dosage on ARS removal at pH 4, 40 ppm dye concentration, and 40 min.

The adsorption capacity is evaluated by considering the solution’s pH, that significantly influences the adsorption mechanism. Influenced by the charge properties of the organic dye and its affinity for the Zn-MOF, pH is selected afterwards^29,30^. The zeta potential of the Zn-MOF was measured to be −16 mV, as shown in figure S1. Adsorption is more efficient in acidic conditions, Because the surface charge of Zn-MOF reaches zero at a pH of 8, the Zn-MOF reached its pHpzc, as demonstrated in Fig. 10. The surface carries a positive charge at pH values below the pHpzc and becomes negative at pH values above it^32^. As a result, the nano Zn-MOF and the anionic dye have a high electrostatic attraction, allowing them to interact more easily. Figure 9 shows that employing anionic dye at a concentration of 40 ppm and pH 4 resulted in a roughly 51% clearance rate. However, it rapidly declined, reaching pH of 8, 8% as a result of the deportation of the sulphonate group. As a result, the dye developed a negative net charge, which was subsequently neutralized by the anionic Zn-MOF nanoparticles.

#### Effect of adsorbent dosage

To uncover the adsorbent dosage’s effect, the amount of nano Zn-MOF was adjusted to a range of 0.01 to 0.05 g/50 mL while keeping the initial dosage of 40 mg/L at pH 4. Figure 10 shows this. When the adsorbent dose was raised from 0.01 to 0.05 g led to an enhancement in removal efficiency, reaching 69% from an initial 51%. This resulted from the Increment in the active adsorption sites on the adsorbent’s surface which in turn enhances the removal percentage when the adsorbent dose is increased^33^.

#### Effect of initial dye concentration

**Fig. 11:**
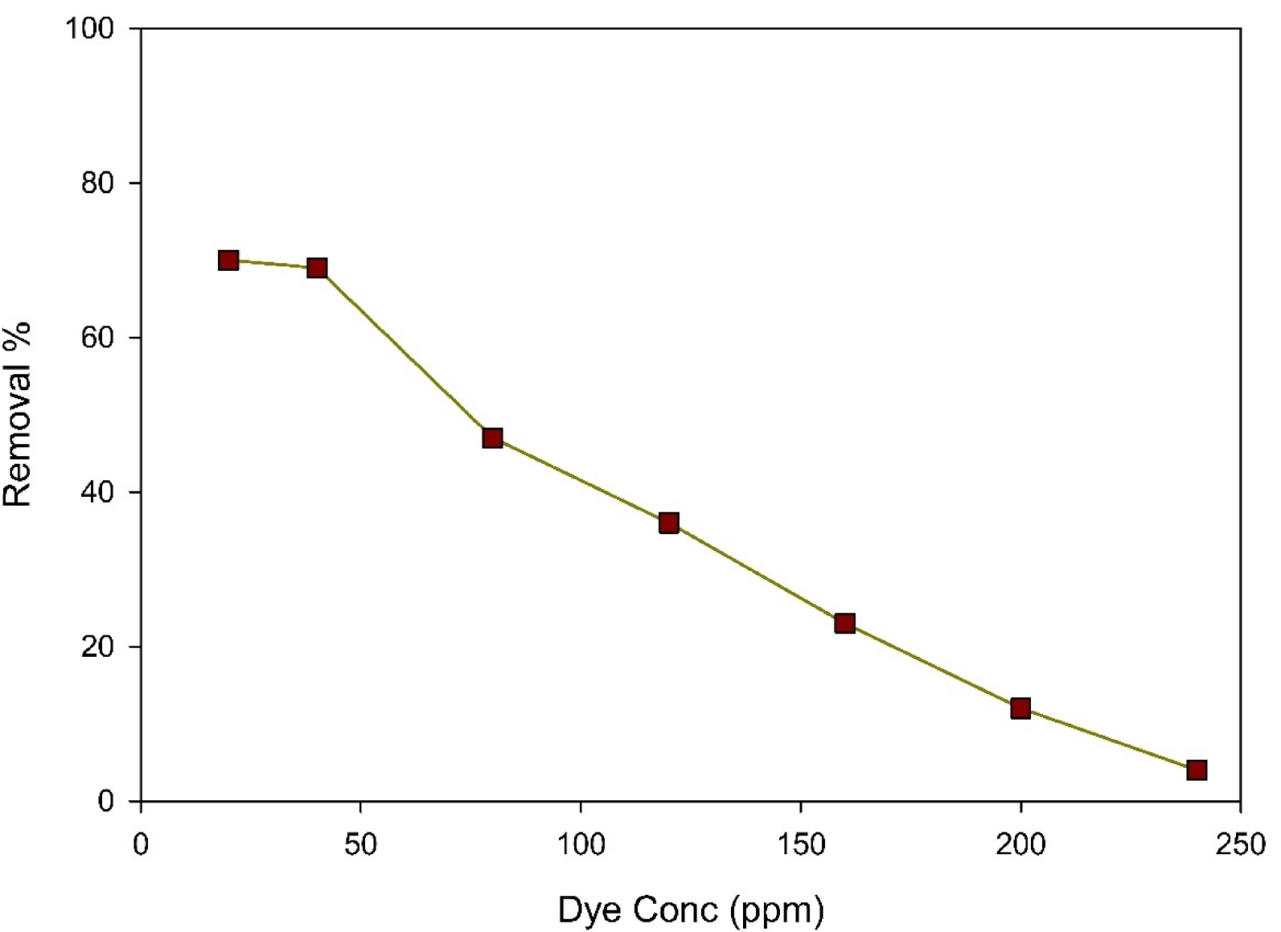
Effect of initial dye concentration on the elimination of ARS at pH 4, 0.05 g Zn-MOF/50 mL, and 40 min.

Accumulation of dye molecules at the solid-liquid interface, which includes mass transfer, is known as dye adsorption. The study sought to examine the impact of the dye’s initial concentration, with the remaining factors kept at a constant rate. Figure 11 shows That reducing the dye’s initial concentration from 40 ppm to 20 ppm leads to an increase in elimination percentage from 69 to 71%. The clearance percentage drops from 69 to 4% as the dye’s initial concentration increases from 40 to 240 ppm. With the rise in initial dye concentration, the lower the removal percentages become since the quantity of active sites available on the adsorbent decreases. Particularly, this becomes more noticeable upon increasing the starting concentration of ARS more than 40 ppm. Another contributing factor to the decrease of the removal rate is resistance to the mass transfer between the different liquid phases (which contain water, the dye, and other components) and the Zn-MOF adsorbent, also known as the solid phase. Nonetheless, the ARS provided a critical impetus to overcome this obstacle in mass transfer. Consequently, the barrier to mass transfer lowered, enhancing dye removal efficiency as the initial concentration declined.

#### Effect of contact time

**Fig. 12:**
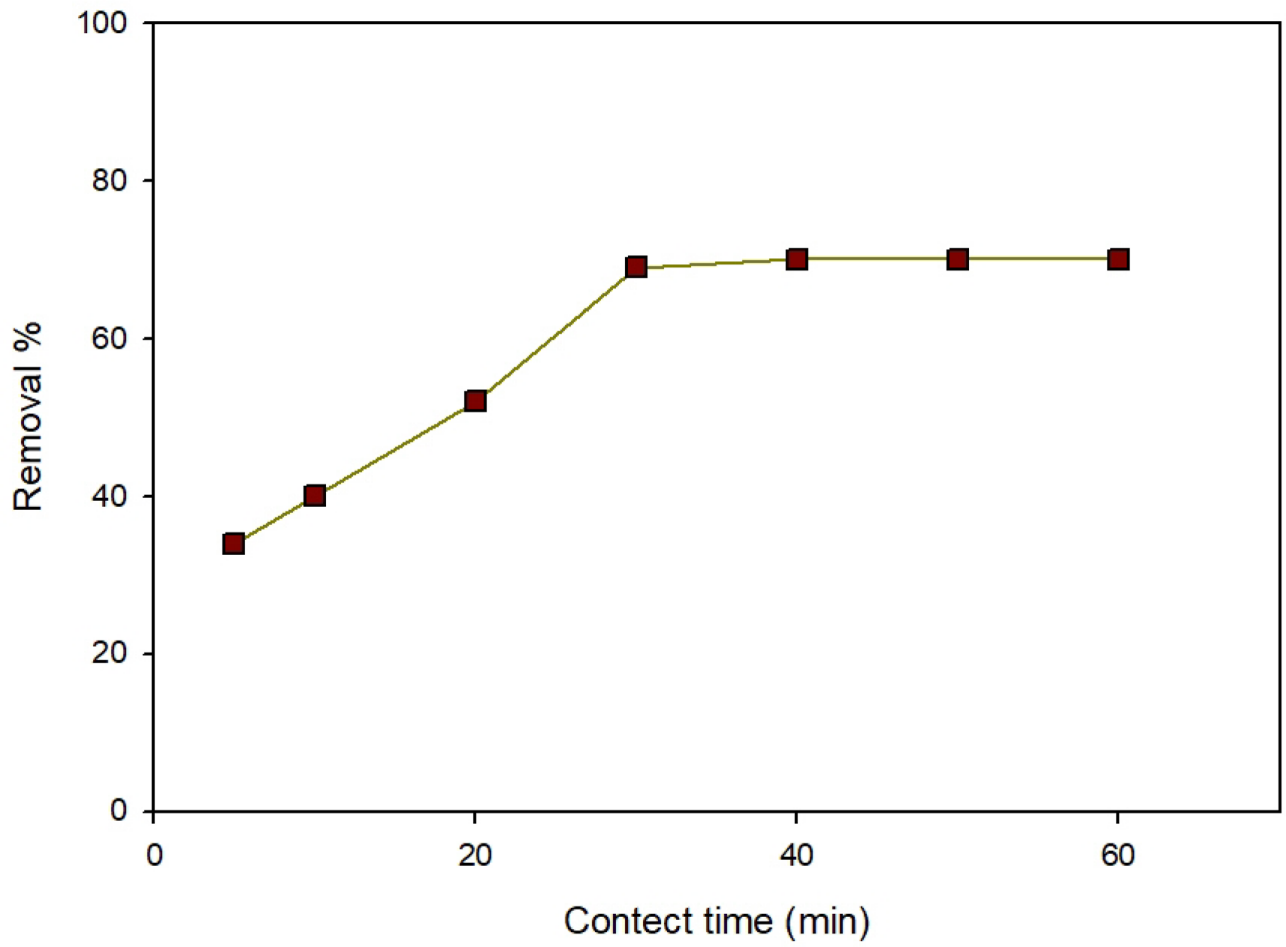
The contact time factor for removing ARS at pH 4 with 0.05 g Zn-MOF/50 mL and 20 ppm dye concentrations.

An efficient system for wastewater treatment carefully considers equilibrium time as a key factor. To understand the adsorption process and determine the effectiveness of an adsorbent, absorption kinetics is essential. Material success as an adsorbent is mainly dependent on its ability to adsorb quickly and quantitatively. Research Was performed to study the contact duration effect on dye adsorption. Time was measured on different intervals from 5 to 60 min. Figure 12 shows 71% clearance after 40 min and remains constant at dye concentration of 40 ppm and pH 4.

#### Adsorption isotherm

Adsorption isotherms were measured for wastewater and water treatment applications using the Freundlich, Temkin, and Langmuir equations^34^. Langmuir isotherm: It is assumed in the Langmuir model that adsorption proceeds at well-defined, uniform sites on the adsorbent, avoiding significant interactions within the adsorbed species. Saturation occurs as a monolayer of molecules becomes adsorbed onto the surface of the adsorbent^34^.

Equation (3) states that:

3 $$q_e=(q_{max}bC_e)/(1+bC_e)$$

Equation 4 describes the linear form:

4 $${C_e}/{q_e} = {\text{ }}1/\left( {{q_{max}}b} \right){\text{ }} + {\text{ }}\left( {1/{q_{max}}} \right){\text{ }}{C_e}$$

**Fig. 13:**
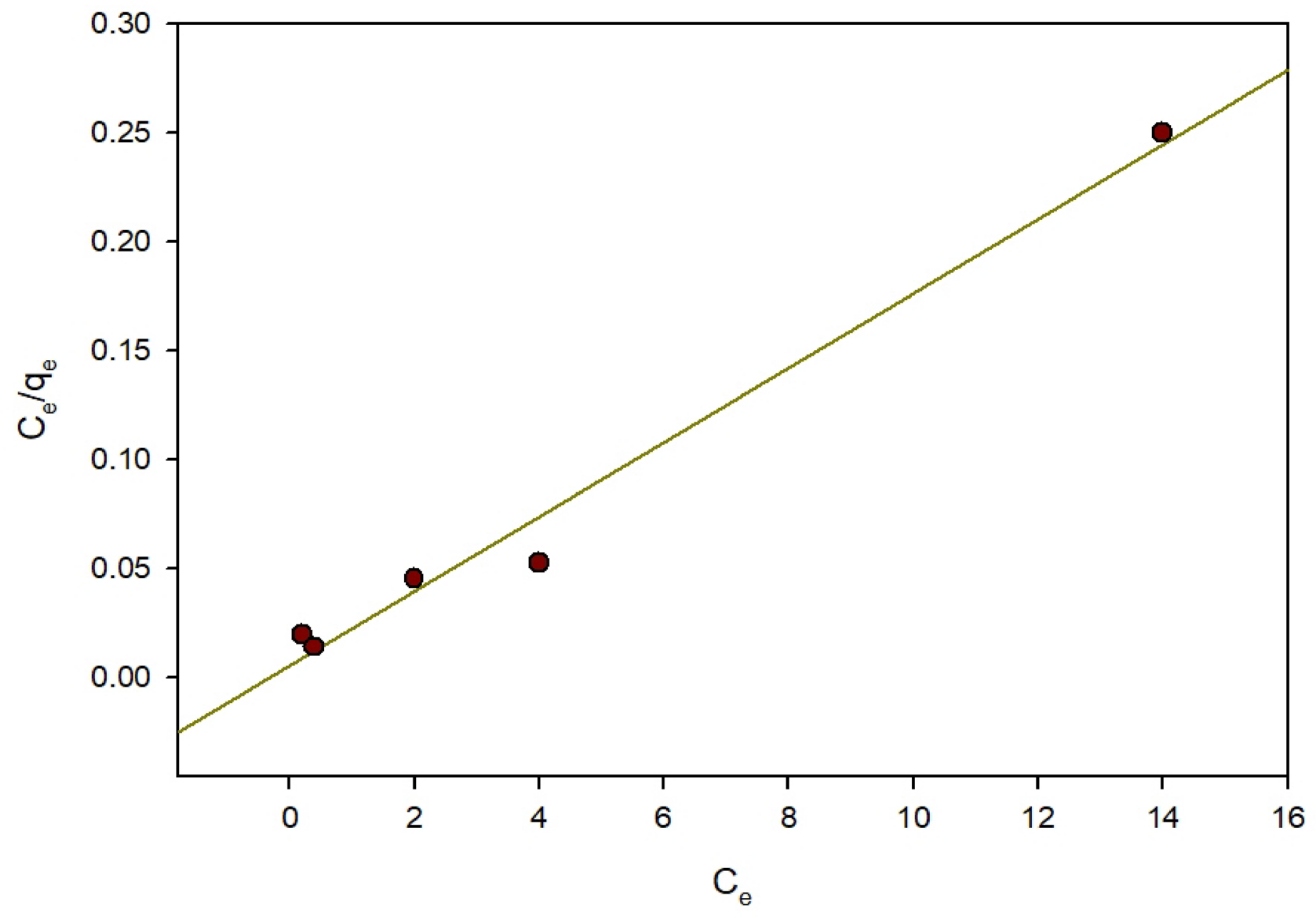
Langmuir adsorption isotherm of ARS on Zn-MOF.

The q_e_ variable indicates the adsorbate mass per gram of sorbent. The highest adsorption capacity, denoted by q_max_, is measured in milligrams per gram. The equilibrium solution’s concentration is C_e_, where milligrams per liter is its measuring unit. Finally, b, with a measuring unit of liters per milligram, reflects the affinity coefficient related to the adsorption energy. The values of q_max_ and b were found after studying the linear relationship between C_e_/q_e_ and C_e_, as indicated in Table 2; Fig. 13. The maximal capacity for adsorption of ARS per gram of Zn-MOF was measured for 59 mg, and b, the Langmuir constant equilibrium, was found to be 2.16. The R^2^ value 0.984 demonstrated a close fit between the Langmuir Isotherm model and the adsorption data. In order to confirm the fundamental Langmuir isotherm’s features, Eq. (5) was used to calculate the separation factor (R_L_) for the maximum adsorbate concentration (C_o_) in mg/L (milligrams per liter).

5 $${R_L} = {\text{ }}1/\left( {{C_o}b{\text{ }} + {\text{ }}1} \right)$$

The R_L_ value Indicates the adsorption isotherm’s favorability, with the process regarded as irreversible when R_L_ = 0, favorable if R_L_ < 1, linear if R_L_ = 1, or unfavorable if R_L_ > 1^32^.

Table 1 demonstrates that the R_L_ values reflect favorable equilibrium adsorption covering concentrations in the range of 10–99 mg/L.

Freundlich isotherm: This model assumes that the sorption system comprises numerous non-uniform layers and surfaces, Including limited adsorption sites and possible energy interactions^34^. Equation (6) represents the mathematical expression of the Freundlich model.

6 $${Q_e} = {\text{ }}{K_f}{C_e}1/n$$

In order to linearize Eq. (7), The subsequent constants and logarithmic values are to be used:

7 $$Log{Q_e} = {\text{ }}log{K_f} + {\text{ }}\left( {1/n} \right)log{C_e}$$

**Fig. 14:**
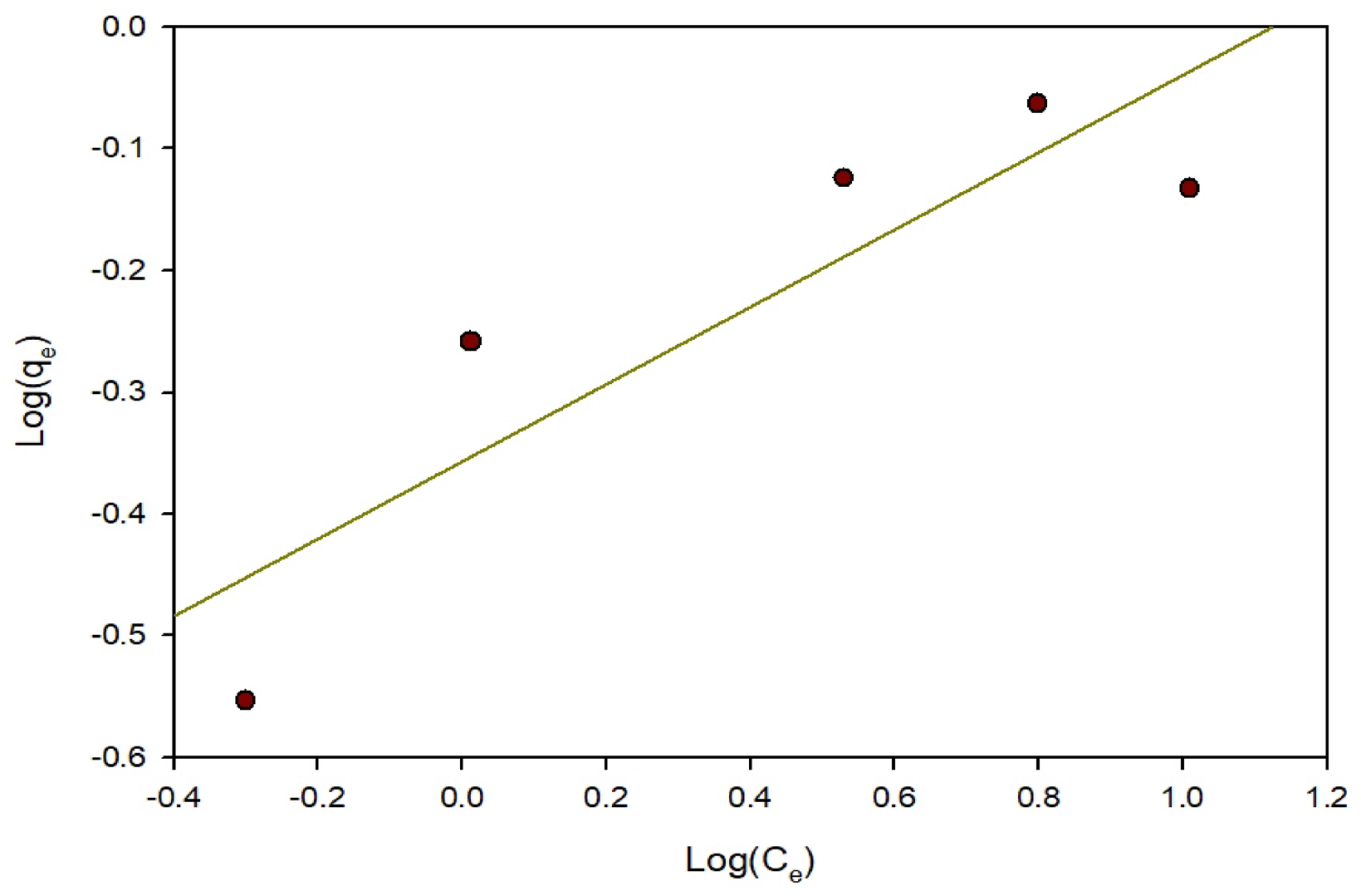
Freundlich adsorption isotherm of ARS on Zn-MOF.

**Table 1:** Values of separation factor (R_L_).

C_0_ (mg/L)	20	40	60	80	100
RL	0.0226	0.0114	0.0076	0.0057	0.0046

The Freundlich model includes the constants K_n__and n., with n indicating Suitability and K_f_ representing the adsorbent capacity. Measurements of 1/n smaller than 1 indicate considerable Adsorption under low-concentration conditions, Whereas the increase in adsorption becomes less pronounced at higher concentrations, and more significant at lower concentrations^36^. Elevated K_f_ values correspond to increased adsorption intensity^36^.

Table 2; Fig. 14 show that ARS adsorption onto Zn-MOF is favorable, with 1/n values ranging from 0 to 1. The surface of Zn-MOF is strongly bound with the dye, with n values in the 1–10 range indicating adsorption onto Zn-MOF.

Temkin isotherm: This isotherm depends on two assumptions. To begin with, it presumes that the adsorption heat across the entire molecular layer decreases linearly with increasing surface coverage, due to interactions between the adsorbent and adsorbate. Furthermore, Adsorption is defined by an even distribution of binding energies that near the highest binding energy level^34^. The Temkin isotherm is frequently applied to represent the uneven distribution of sorption heat^37^.

8 $${Q_e} = {\text{ }}\left( {RT/b} \right)lnA{\text{ }} + {\text{ }}\left( {RT/b} \right)ln{C_e}$$

**Fig. 15:**
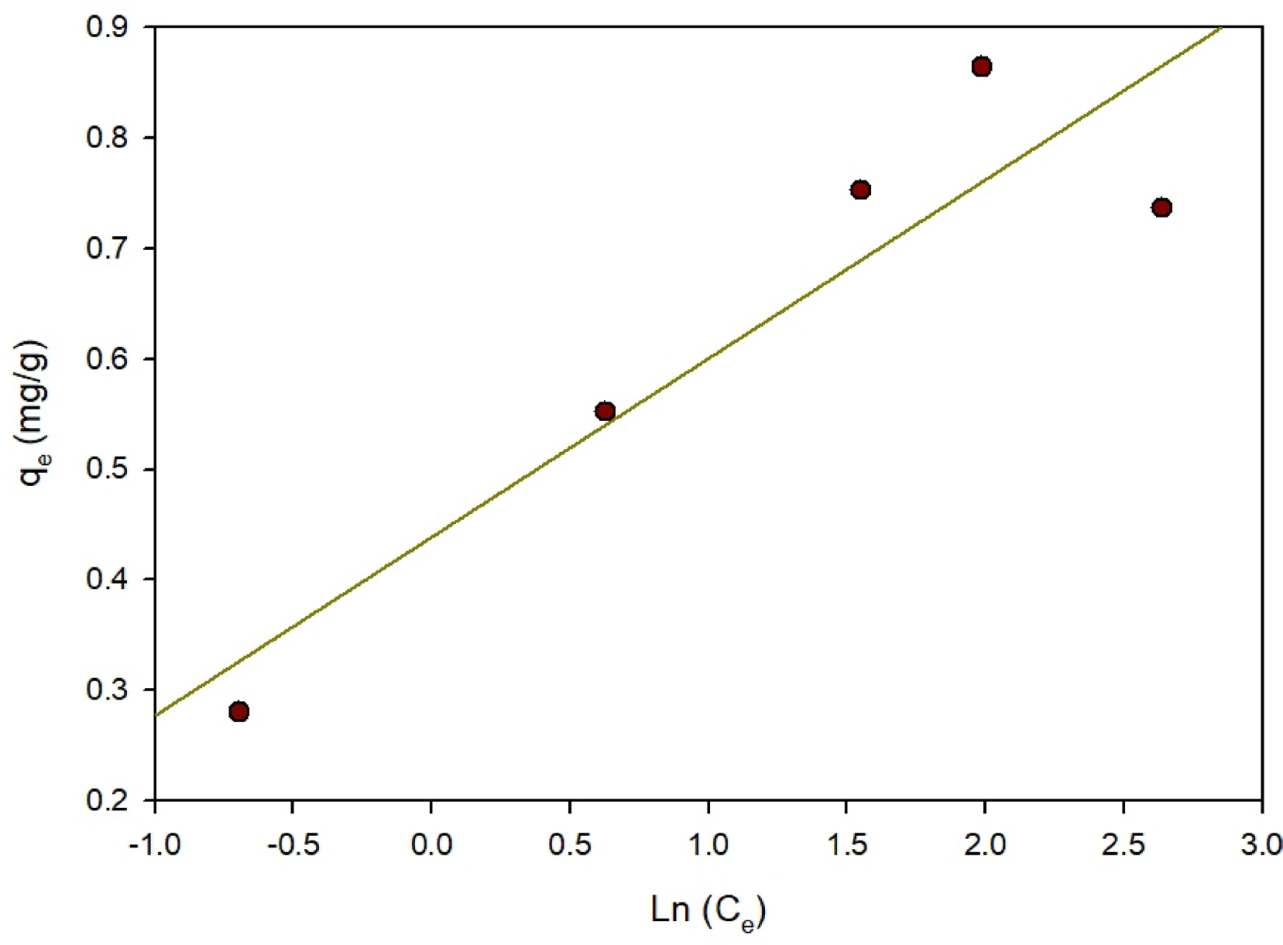
Temkin adsorption isotherm for ARS on Zn-MOF.

The equilibrium adsorption constant, A (L/mol), represents the highest binding energy. The heat constant for adsorption is B = RT/b constant linked along with the sorption heat (J/mol) computed from a Temkin plot was constructed by plotting q_e_ against lnC_e_; with R representing the universal gas constant (8.314 J/mol K); and T is the temperature (298 K)^38^. Figure 15 shows that The Temkin isotherm was represented by a linear relationship. Table 2 shows that the slope, B = RT/b, yields the Temkin isotherm constant (b), whereas the intercept, (RT/b) lnA, yields the equilibrium constant derived from the Temkin isotherm (A). According to the R^2^ results, the Langmuir isotherm is more appropriate for ARS adsorption on Zn-MOF, with a correlation coefficient (R^2^) of 0.984, which demonstrates that the Langmuir adsorption model effectively replicates the experimental findings and indicates that the adsorption process took place as a monolayer^39^. This means that the sorption capabilities are determined by the adsorbent’s surface, The molecular organization on the surface, and the nature of interactions between ARS and the adsorbent.

**Table 2:** Langmuir, Freundlich, Temkin and R^2^ values of ARS adsorption on Zn-MOF.

Isotherm	Results
Langmuir isotherm	q_max_ = 59 mg/g
	b = 2.16 L/mg
	R^2^ = 0.984
Freundlich isotherm	K_f_= 0.48 (mg/g)
	1/*n* = 0.32
	R^2^ = 0.77
Temkin isotherm	B = 0.164 J/mol
	b = 15.1
	A = 0.28 L/mol
	R^2^ = 0.81

#### Adsorption kinetics

Kinetics of ARS elimination were investigated in this study by Zn-MOF using the pseudo-first and second order kinetics. According to equation number (9), the pseudo-first order module indicates the amount of adsorbed adsorbate on the surface of the adsorbent over time (t). The reaction is consistent when one concentration or more influences the reaction rate significantly. It defines adsorption on adsorbent surfaces that are heterogeneous.

9 $$Log\left( {{q_e} - {\text{ }}{q_t}} \right){\text{ }} = {\text{ }}log{\text{ }}{q_e} - \left( {{k_1}/2.303} \right)t$$

**Fig. 16:**
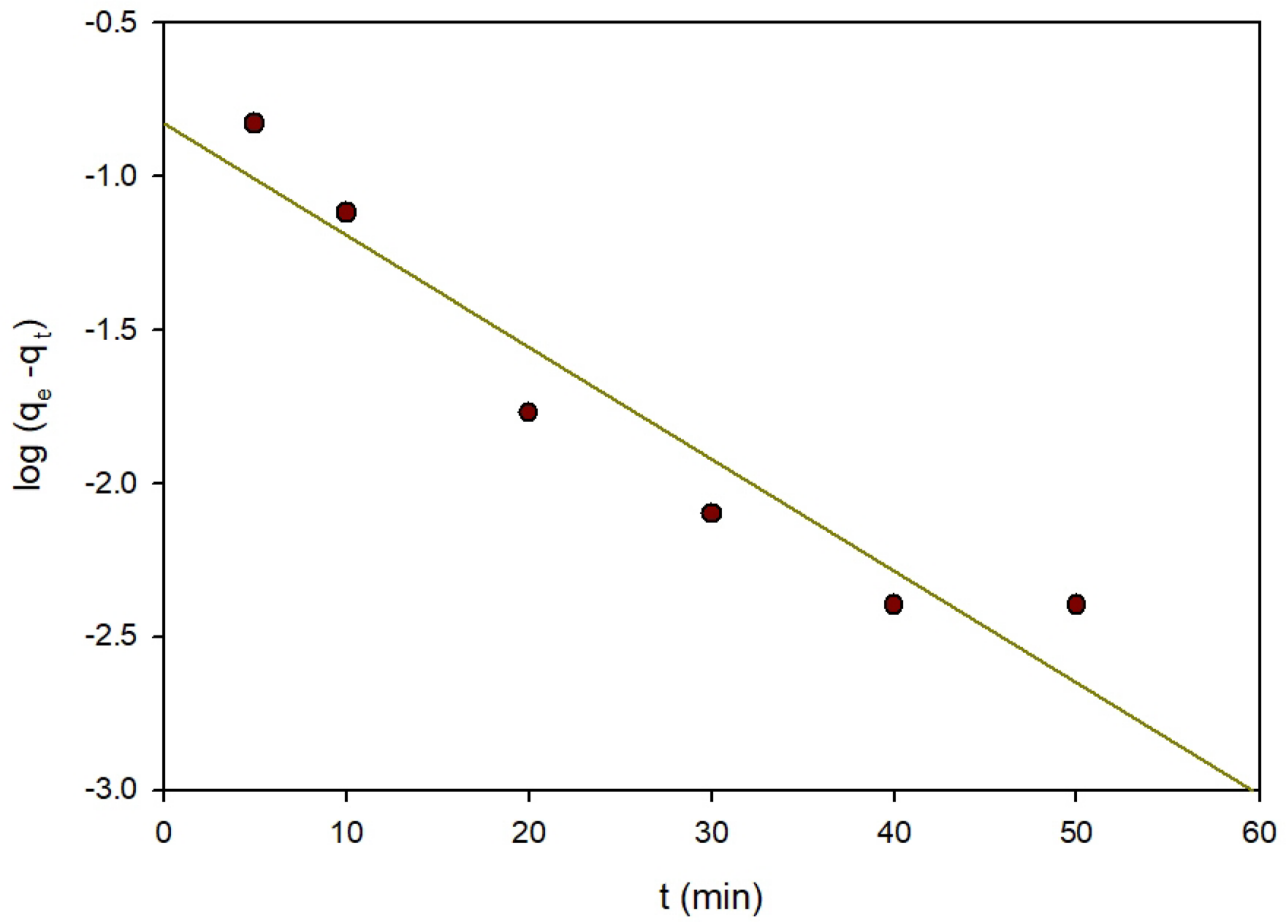
Pseudo-first order kinetics of the removal of ARS.

Alternatively, the pseudo-second order module was created to closely mimic the real reaction. According to^40^, the slowest process is chemisorption, which can be described in the following Eq. (10): t/q_t_ = (1/q_e_)t + 1/k_2_q_2_ (10).

10 $$t/{q_t} = {\text{ }}\left( {1/{q_e}} \right)t{\text{ }} + 1/{k_2}{q_2}$$

**Fig. 17:**
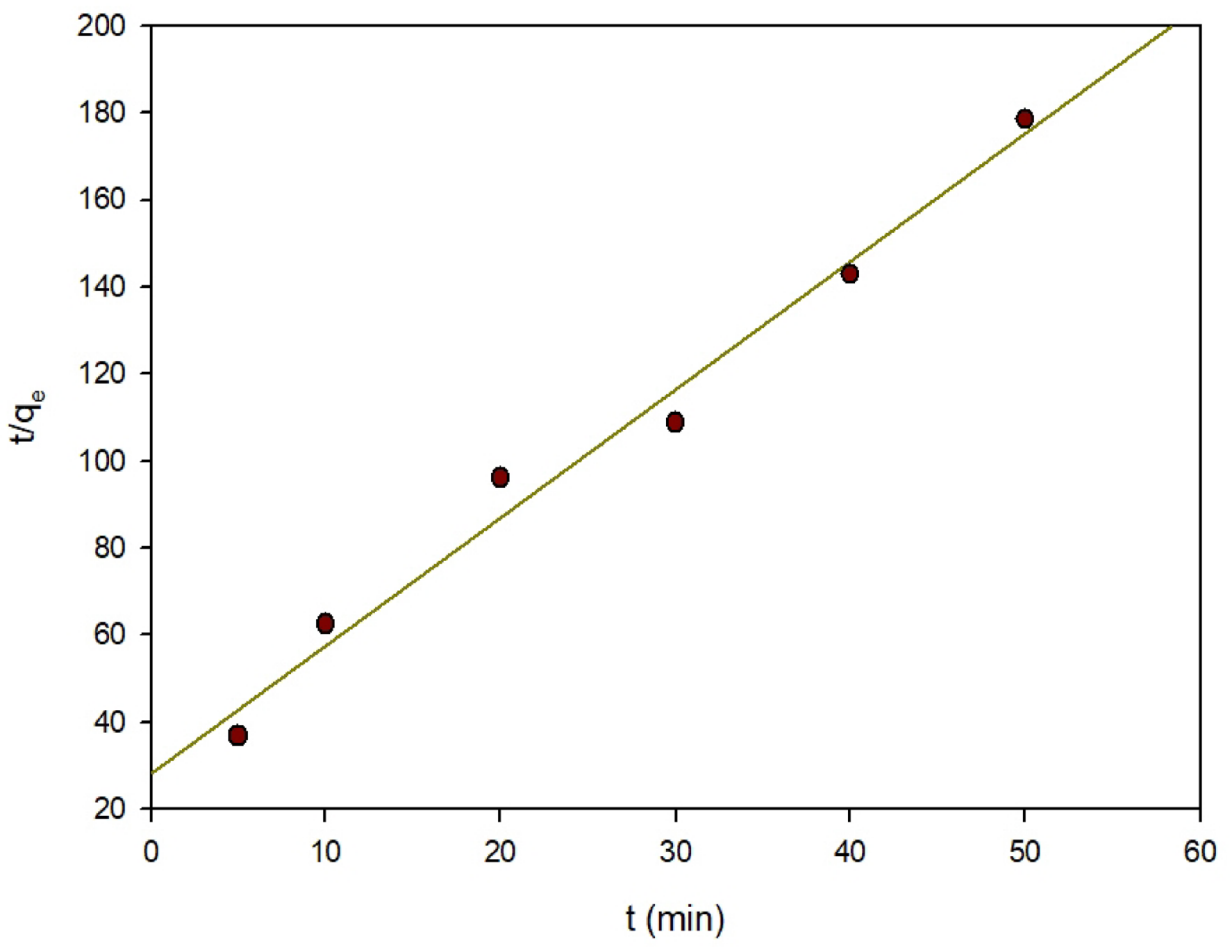
Pseudo-second order kinetics of the removal of ARS.

Where k_1_ and k_2_ are respectively the first and second-order rate constants, and q_e_ and q_t_ respectively are the quantities of dyes adsorbed on the surface of the Zn-MOF at equilibrium and time (t) in mg/g. The previously specified parameters are determined and listed as shown in Table 3. The pseudo-first-order kinetic model shows low correlation values (R^2^ = 0.894) as in Fig. 16. Furthermore, there was a perceptible difference in the q_e_ (equilibrium adsorption capacity) regarding the actual and theoretical results, demonstrating the ineffectiveness of the pseudo-first order model to the experimental data. Pseudo-second order kinetics resulted in a linear fit with high correlation coefficients (R^2^ = 0.998), as illustrated in Fig. 17. As for pseudo-second order kinetics, the theoretical q_e_ values are consistent with the experimental observations.

**Table 3:** The kinetic parameters for ARS adsorption on Zn-MOF.

Kinetic model	Parameter
Pseudo-first order	q_e_ = 0.158 mg/g
	K_1_= (min-1)
	R^2^ = 0.894
Pseudo-second order	q_e_ = mg/g
	K_2_ = (min-1)
	R^2^ = 0.998

#### Regeneration

To validate Zn-MOF’s environmental friendliness and cost-effectiveness, the regeneration process must be carried out. After adsorption, the adsorbent is washed with a 0.01 mol/L solution of HCl and NaOH and rinsed using distilled water to complete the desorption process. After three cycles with Zn-MOF, the active sites are reduced, resulting in around 60% removal effectiveness (Fig. 19). The data revealed that Zn-MOF was highly cost-effective.

### Comparison between different types of adsorbents

Table 4 shows that Zn-MOF outperforms other materials in terms of ARS eradication speed and effectiveness. Additionally, its efficiency at lower dosages than the durian husk material was discovered. The obtained results can be attributed to these nanoparticles’ specific properties, as described in cited publications.

**Table 4:** Comparison between different types of adsorbents for removal of ARS.

Adsorbent	Treatment	Adsorption Isotherm	Contact Time	pH	Removal Percentage	Dosage (g/L)	Initial Conc. (ppm)	Refs.
Citrullus lanatus peels	Adsorption	Temkin	40 min	2 to 4	89.44%	0.3	15	^41^
sheep wool	Adsorption	Langmuir and Freundlich	90 min	2	93.2%	8	-	^42^
maghemite iron oxide (γ-Fe_2_O_3_) nanoparticles	Adsorption	Langmuir	60 min	11	95%	-	24	^43^
[Ca(BDC) (H_2_O)_3_]	adsorption	Langmuir-Freundlich		6	69.97%	0.002	379	^44^
UiO-66	adsorption	Langmuir	-	2	-	0.0248	11.82	^45^
Zn-MOF	Adsorption	Langmuir	40 min	4	71%	1	20	This work

**Fig. 18:**
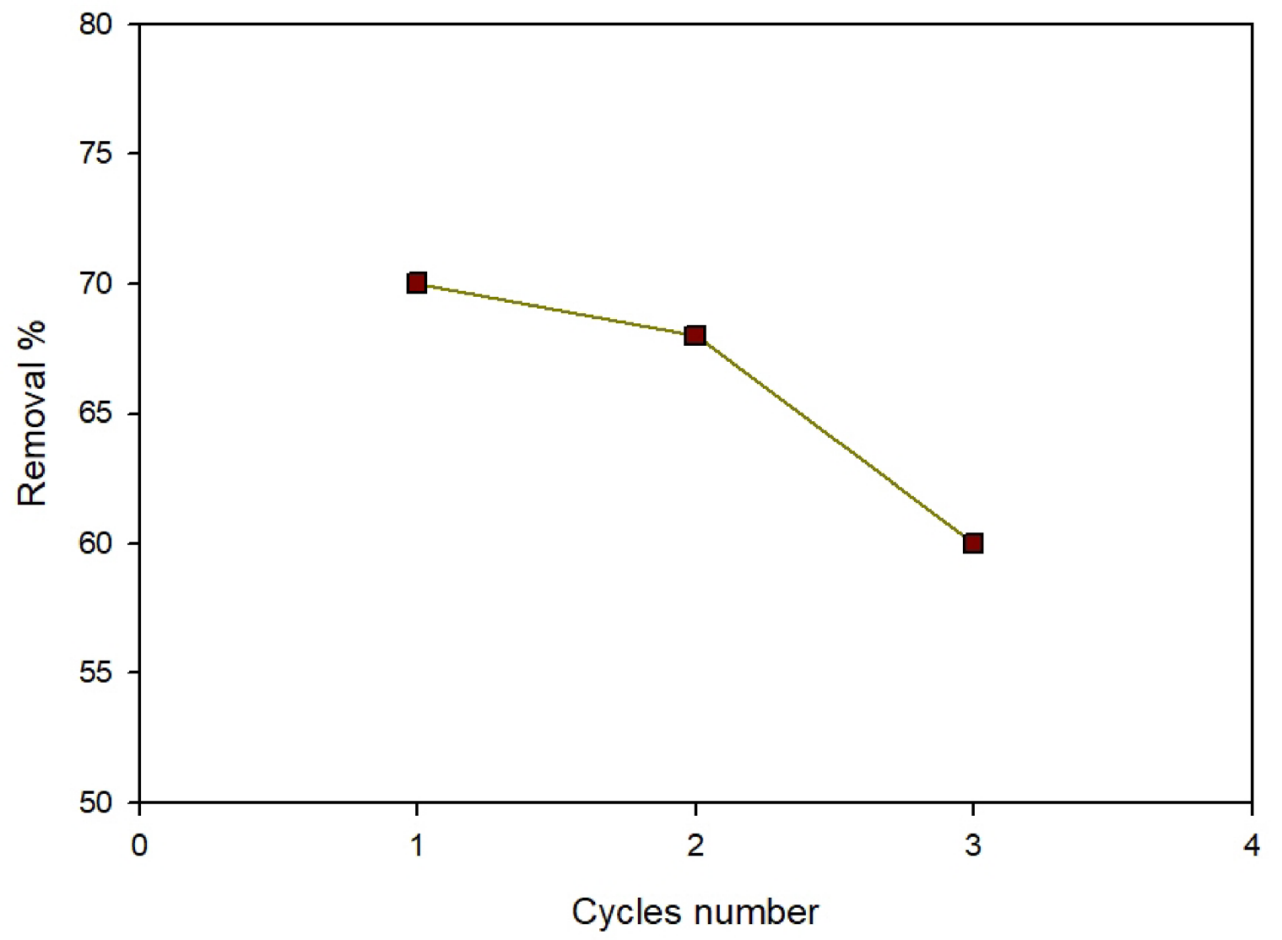
Cycles number of regeneration.

## Conclusion

Zn-MOF was produced using solvothermal technique and studied with FT-IR, XRD, SEM, EDX, and BET. Zn-MOF (0.05 g) was utilized as an adsorbent for ARS at a pH 4 and initial dye concentration of 20 ppm. Within 40 min and at room temperature, the elimination rate reached about 71%. The experimental data strongly matched the Langmuir model with the highest regression coefficient of R^2^ = 0.984. The q_max (_maximum adsorption capacity) was 59 mg/g. The kinetic data indicated that a pseudo-second-order mechanism governs the adsorption process. Studies in regeneration showcased the potential of Zn-MOF after being used for three consecutive cycles with high removal efficiency. Zn-MOF’s features increased their potentiality in wastewater treatment for eradicating ARS.

## Supplementary Information

Below is the link to the electronic supplementary material.

Supplementary Material 1

## Data Availability

Data available upon request from the corresponding author (asalah@sci.cu.edu.eg).
