# Supplementary material for: Synthesis and characterization of novel zinc–organic framework for the effective removal of Alizarin Red S
Source: Sci Rep. 2025 Dec 8;15:43275. doi: 10.1038/s41598-025-29056-5 (PMC12686488; doi:10.1038/s41598-025-29056-5)
Supplement: Supplementary file 1 — Supplementary Material 1 [file 41598_2025_29056_MOESM1_ESM.docx]

**Synthesis and Characterization of Novel Zinc–Organic Framework for the Effective Removal of Alizarin Red S**

**Mohamed A. Abdelwahab** ^a^**, Abdelhamid M. Abdelhamid** ^b^**, Ashraqat M. Abdelhamid** ^b^**, Sohaila Abdelhady** ^b^**, Soad Auf** ^b^**, Hala A. Awad** ^b^**, Raghad M. Abdelkader** ^b^**, Jana Ghanem** ^b^**, Malak H. Mohamed** ^b^**, Mariam Gamaleldin** ^b^**, Saher A. Ali** ^b^**, Gehad G. Mohamed** ^a,c^**, Ayman S. Eliwa ^a*^, Maha Alhelf** ^b,d^

^a^ Chemistry Department, Faculty of Science, Cairo University, 12613 Giza, Egypt

^b^ Biotechnology School, Nile University, 12588, Giza, Egypt

^c^ Nanoscience Department, Basic and Applied Sciences Institute, Egypt-Japan University of Science and Technology, New Borg El Arab, Alexandria, 21934, Egypt

^d^ Medical Biochemistry and Molecular Biology Department, Faculty of Medicine, Cairo University, Cairo, Egypt

^*^Corresponding author

Ayman S. Eliwa: [asalah@sci.cu.edu.eg](mailto:asalah@sci.cu.edu.eg)


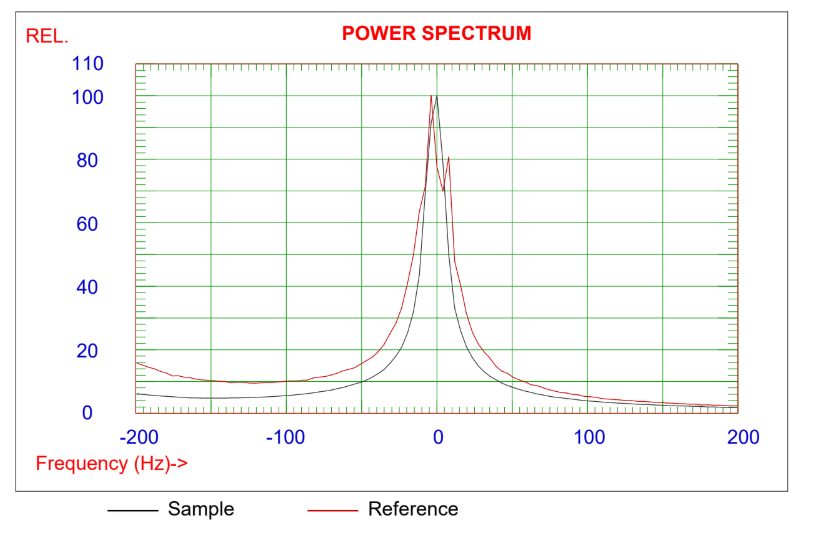


Supplementary figure S1. Zeta potential curve for Zn-MOF.


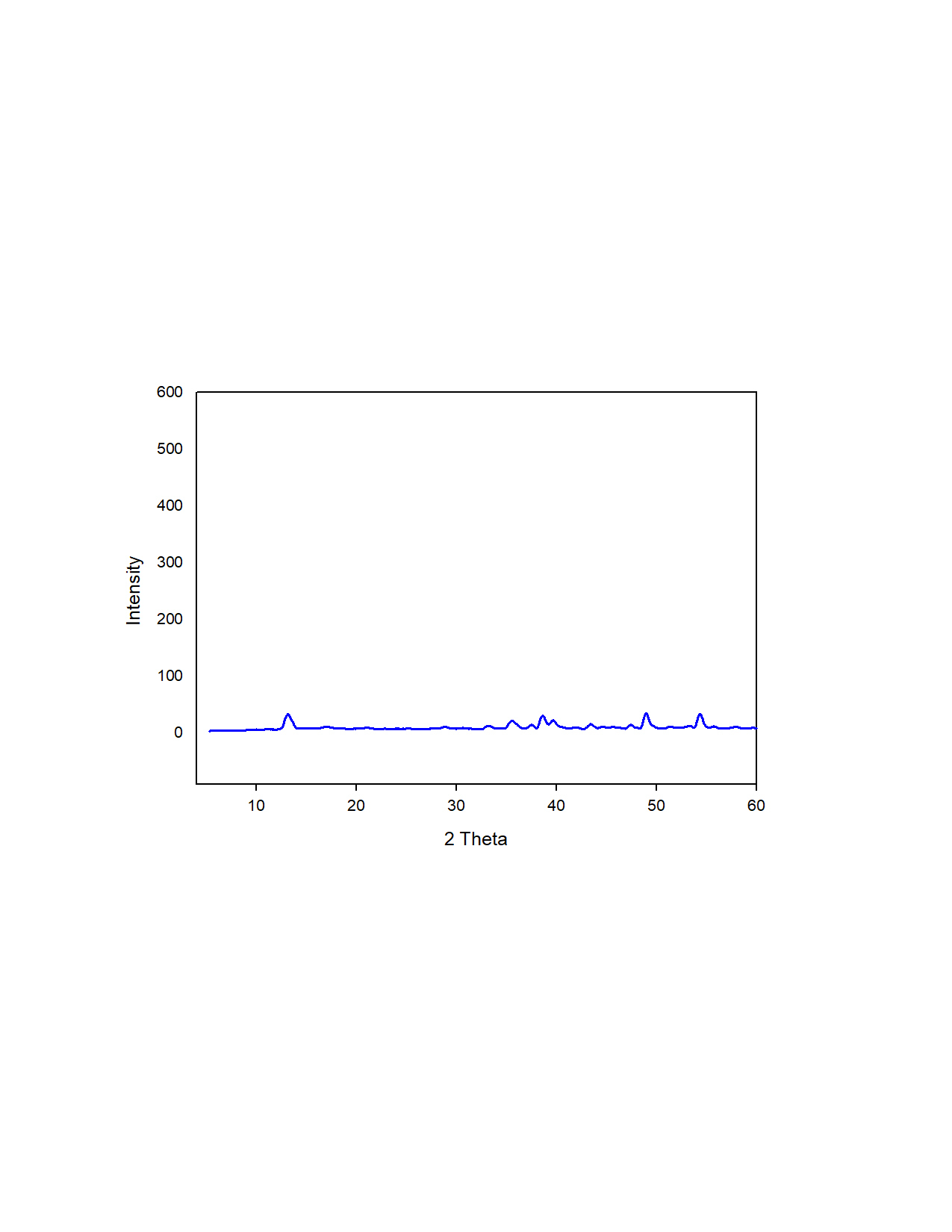
Supplementary figure S3. Powder X-ray diffraction for Co-MOF after application.
